# Different protein expression associated with chemotherapy response in oropharyngeal cancer according to HPV status

**DOI:** 10.1186/1471-2407-14-824

**Published:** 2014-11-07

**Authors:** Min-Jee Kim, Myung-Seo Ki, Karham Kim, Hyun-Jeong Shim, Jun-Eul Hwang, Woo-Kyun Bae, Ik-Joo Chung, Dong-Hoon Lee, Joon-Kyoo Lee, Tae-Mi Yoon, Sang-Chul Lim, Woong-Ki Chung, Jae-Uk Jeong, Hoi-Soon Lim, Yoo-Duk Choi, Sang-Hee Cho

**Affiliations:** Department of Hematology-Oncology, Chonnam National University Hwasun Hospital, 322 Seoyangro, Hwasun, Jeollanamdo, 519-763 Republic of Korea; Department of Otolaryngology-Head and Neck Surgery, Chonnam National University Hwasun Hospital, Jeollanamdo, Republic of Korea; Department of Radiation Oncology, Chonnam National University Hwasun Hospital, Jeollanamdo, Republic of Korea; Department of Dentistry, Chonnam National University Hwasun Hospital, Jeollanamdo, Republic of Korea; Department of Pathology, Chonnam National University Hwasun Hospital, Jeollanamdo, Republic of Korea

**Keywords:** Oropharyngeal cancer, HPV, p16, Chemotherapy, p53, Beta tubulin, bcl-2

## Abstract

**Backgound:**

Oropharyngeal cancer (OPC) associated with human papilloma virus (HPV OPC) shows better treatment outcomes than non-HPV OPC. We investigated the expression of p53, β-tubulin, bcl-2 and ERCC 1, which are well-known biomarkers to predict the chemotherapy response, according to HPV status in OPC patients.

**Methods:**

Patients who treated with at least 2 cycles of induction chemotherapy followed by concurrent chemoradiotherapy for locally advanced oropharyngeal cancer were reviewed. HPV PCR and immunohistochemical stain was done in paraffin embedded tumor tissue and evaluated the relation with the chemotherapy response and survival outcomes according to HPV status.

**Results:**

Seventy-four patients were enrolled for this study and all patients received induction chemotherapy with docetaxel, 5-FU and cisplatin. After induction chemotherapy, complete response (CR) was shown in 22 patients (30%) and partial response (PR) in 46 patients (62%). HPV + was detected in 21 patients (28%), while 35 patients (47%) showed p16+ expression by IHC analysis. p16 positive patients showed better overall response, PFS and OS than p16 negative patients. p53 and class III beta-tubulin expression were significantly higher in HPV- and p16- than HPV + and p16+ patients. Conversely, bcl-2 expression was greater in HPV + or p16+ than HPV- or p16- patients. ERCC1 expression did not differ significantly according to HPV status. In multivariate analyses, early T stage (*p* = 0.036) and good PS (PS 0) (*p* = 0.029) showed a better 3Y-PFS rate, and low p53 expression (*p* = 0.012) and complete response after induction chemotherapy (*p* = 0.026) were highly associated with 3Y-OS rate. Low expression of p53 and p16 positive patients showed significantly prolonged OS than others (*p* = 0.010).

**Conclusion:**

P53, class III beta-tubulin and bcl-2 were differently expressed in OPC according to HPV status and present study suggested the underlying mechanism of better response to chemotherapy in case of HPV OPC than non-HPV OPC. Among these biomarkers, p53 is the strongest prognostic marker in OPC and p53 in addition to p16 support the rationale to study of de-escalation strategy for OPC.

## Background

Among the head and neck cancers, which are well known for their heterogeneity, oropharyngeal cancer (OPC) has been reevaluated because its pathogenesis is associated with human papilloma virus (HPV). The incidence of oropharyngeal squamous cell carcinoma has increased over the past few decades in western countries as well as in Asia including Korea, particularly in younger adults and non-smokers [1–3]. Reliable evidence suggests that OPC associated with HPV (HPV OPC) has a better prognosis than non-HPV-associated OPC (non-HPV OPC) [4–6]. HPV OPC may have different epidemiological and histopathological characteristics than other head and neck cancers, which are usually associated with smoking and alcohol use [7, 8]. The molecular profiles of HPV OPC are characterized by p53 degradation, retinoblastoma RB pathway inactivation by E6 and E7 oncoprotein overexpression, respectively and p16 upregulation [9]. In contrast, non-HPV OPC is associated with smoking-induced multistep carcinogenesis, such as frequent TP53 mutations and p16 impairment [10]. HPV OPC responds better to chemotherapy and radiotherapy, which can be in part explained by non-mutant TP53 [11, 12], absence of field cancerization related to tobacco use and functional p53-mediated apoptotic pathways [13] unlike non-HPV OPC occurring in younger patients, which has fewer comorbidities and a better performance status. However, biomarkers for chemotherapy response in OPC according to HPV status have not been identified.

In locally advanced or unresectable head-and-neck cancers, docetaxel, cisplatin and 5-FU (DCF) has been used as an induction chemotherapy, and showed a high response rate and improved survival [14, 15]. p53 is a well-known prognostic factor and predictive marker of a response to chemotherapy in head-and-neck squamous cell carcinoma (HNSCC), including OPC [16, 17]. In addition, the effects of class III beta-tubulin on taxane and ERCC1 on platinum-based chemotherapy have been investigated in various tumors, including those of the stomach and lung [18–20]. High expression of bcl-2 is reportedly a favorable prognostic factor [21] in head-and-neck cancers. Therefore, in this study we evaluated the expression of these biomarkers according to HPV status and the ability to predict the response to chemotherapy in OPC patients treated with induction chemotherapy using DCF followed by concurrent chemoradiotherapy (CCRT). We also identified the optimal surrogate marker to predict the prognosis of HPV OPC.

## Methods

Patients (≥18 years of age) diagnosed with locally advanced OPC between June 2004 and December 2011 were reviewed retrospectively. Inclusion criteria for this study were a diagnosis of squamous cell carcinoma, tumor stage III to IV according to the American Joint Committee on Cancer Staging [22], treatment with at least two cycles of induction DCF chemotherapy with or without following CCRT, evaluation of the response to induction chemotherapy, paraffin-embedded tumor tissue available at diagnosis and informed consent provided, a Karnofsky performance status (KPS) ≥70 at diagnosis, and sufficient organ function to undergo chemoradiotherapy (CRT). Exclusion criteria included disease location other than the oropharynx, other confirmed or suspected malignancies or a cancer other than squamous carcinoma. Data regarding patients’ characteristics, chemotherapy response, progression-free survival (PFS), and overall survival (OS) were obtained from medical records.

Induction chemotherapy was administered using docetaxel (70 mg/m^2^ on Day 1), cisplatin (75 mg/m^2^ on Day 1) and 5-FU (1000 mg/m^2^ on Day 1–4) repeated every 3 weeks for up to three cycles. Chemotherapy was discontinued in patients who did not respond to induction chemotherapy, and salvage surgery or radiotherapy was performed. After induction chemotherapy, definitive treatment such as CCRT or surgery was performed based on a consensus of the multidisciplinary head-and-neck cancer team. Standard radiotherapy was started within 4 weeks of induction chemotherapy completion and wide treatment fields were planned to encompass the primary tumor site and neck area involved. Treatment consisted of a single daily isocentric external-beam megavoltage irradiation administered at 1.8 to 2.0 Gy per fraction. The primary tumor and affected neck area received 65 to 70 Gy. A minimum of 45 Gy was delivered bilaterally to clinically uninvolved neck areas and supraclavicular regions. Definite irradiation was scheduled with concurrent administration of cisplatin in all patients, except for those with a performance status or residual toxicity precluding the co-administration of chemotherapy. Cisplatin was administered every 3 weeks at a dose of 100 mg/m^2^ depending on creatinine clearance. Cisplatin administration was delayed if evidence of dehydration, renal toxicity, neurotoxicity or ototoxicity was present. For patients with grade 3/4 mucositis or dysphagia, radiation therapy was delayed until recovery to less than grade 2 toxicity. Patients with disease progression or for whom definitive treatment was not available received further chemotherapy as palliative care.

The response evaluation was based on the Response Evaluation Criteria in Solid Tumors (RECIST 1.1) and was assessed after induction chemotherapy and 8 weeks later, following completion of CRT. For all patients with a complete response (CR) on physical examination and CT or MRI scan, a [^18^ F] fluorodeoxyglucose positron emission tomography (^18^ F-FDG-PET) scan was performed for confirmation at 1 month after confirmation of CR. Upon completion of treatment, patients were followed-up monthly by physical examination and CT or MRI scanning was performed every 4 months for 2 years and twice annually thereafter until disease progression. This study was approved by the Institutional Review Board of Chonnam National University Hwasun Hospital (CNUHH-2014-041).

### HPV detection and genotyping

Oropharyngeal cancer tissues were reviewed by the pathologist and tumor cells were identified by light microscopic examination. Before genomic DNA extraction, 20 μm paraffin sections were incubated from the formalin-fixed paraffin-embedded tissue using the QIAGEN Multiplex PCR kit (QIAGEN, Germany) with specific HPV primer sets. All multiplex PCR reactions were followed by manufacturer instructions. Each PCR was carried out in a DNA thermal cycler (PCR thermal cycler Dice, Takara, Japan) with the following conditions: denaturing at 95 for 15 min; 10 cycles of 30 s at 94, 90 s at 63, and 90 at 72; and extension at 72 for 10 min. PCR products were analyzed by electrophoresis on a 2% agarose gel containing ethidium bromide. HPV type was designated based on the band pattern. In cases where band interpretation was not clear, an additional PCR amplification with specific primers was performed to confirm. Selected PCR amplified fragments were cloned into pCR 2.1 vector (Invitrogen, USA), each cloned product was sequenced to confirm fragment identity. Primers for each reaction were followed as previous report [23].

### Immunochemistry

Automated immunohistochemical staining was performed using the Bond-max system (Leica Microsystems, Bannockburn, IL), which is able to process up to 30 slides at a time. Slides carrying the 2-μm thickness tissue sections cut from paraffin-embedded tissue blocks were labeled and dried for 1 hour at 60°C. These slides were then covered by Bond Universal Covertiles (Leica Microsystems) and placed into the Bond-max instrument. All subsequent steps were performed by the automated instrument according to the manufacturer’s instructions (Leica Microsystems), in the following order: (1) deparaffinization of the tissue slides with Bond Dewax Solution (Leica Microsystems) at 72°C for 30 minutes; (2) heat-induced epitope retrieval (antigen unmasking) with Bond Epitope Retrieval Solution 1 (Leica Microsystems) for 20 minutes at 100°C; (3) peroxide block placement on the slides for 5 minutes at ambient temperature; and (4) incubation with P53 (1:1200 dilution, DO-7, DAKO, Denmark), Class III beta-tubulin (1:1000 dilution, TUJI, Convance, Princeton, NJ. USA), ERCC1 (1:1000 dilution, 8 F1, Abcam, Cambridge, UK), Bcl-2 (1:500 dilution, 124, DAKO, Denmark), and p16 (1:50 dilution, G175-407, BD PharMingen, Sandiego, CA, USA) primary antibody for 15 minutes at ambient temperature; (5) incubation with Post Primary reagent (Leica Microsystems) for 8 minutes at ambient temperature, followed by washing with Bond Wash solution (Leica Microsystems) for 6 minutes; (6) Bond Polymer (Leica Microsystems) placement on the slides for 8 minutes at ambient temperature, followed by washing with Bond Wash and distilled water for 4 minutes; (7) color development with DAB (3,3’-diaminobenzidine tetrahydrochloride) chromogen for 10 minutes at ambient temperature; and (8) hematoxylin counterstaining for 5 minutes at ambient temperature, followed by mounting of the slides. Normal human serum served as a negative control.

### Evaluation of immunohistochemical staining

The evaluation of all immunohistochemical staining was done as a blind assessment and independently by two authors. Any discordant findings between the two observers were settled using a conference microscope. Discordance between the two examiners never exceeded 10%. For p53 and ERCC1, nuclear staining was regarded as positive, but, for Bcl-2 and class III beta-tubulin, cytoplasmic staining was positive. Assessment of staining of p53, ERCC1, Bcl-2 and class III beta-tubulin was evaluated based on the staining intensity (SI). SI was scored on a scale of 0–3 (0 ¼ negative staining, 1 ¼ weakly positive staining, 2 ¼ moderately positive staining, and 3 ¼ strongly positive staining). The intensity of staining was evaluated according for to the maximum intensity among positive cells. Tumors were categorized as high expression (SI: 2, 3) or low expression (SI: 0, 1). The immunoreactivity of p16 was evaluated as described previous method [24]. Positive was defined as strong and diffuse nuclear and cytoplasmic staining in 80% or more of the tumor cells.

### Statistical analysis

Association analyses among HPV status, protein expression, and clinicopathological parameters were performed using the chi-square test and Fisher’s exact test. Survival curves (OS and PFS) were calculated using the Kaplan–Meier method and curves were compared using the log-rank test. OS was defined as the period from the time of diagnosis to the time of death or last follow-up. PFS was defined as the time from treatment initiation to tumor progression. Univariate analysis was performed using Kaplan-Meier method and log-rank test. All variables from univariate analysis showing *p* values <0.1 were incorporated in the multivariate Cox hazard regression model with a step-wise forward procedure. SPSS version 20.0 (IBM, Inc., Chicago, IL, USA) was used for statistical analyses. A *p*-value <0.05 was considered to indicate statistical significance.

## Results

### Patient characteristics according to HPV status

Eighty-seven patients were treated with induction chemotherapy followed by CRT for locally advanced OPC between June 2004 and June 2011. Of these, 10 patients had no available tissue and 3 patients received one cycle of induction chemotherapy without follow-up examination; therefore, 74 patients were enrolled in this study. Five patients received two cycles of induction chemotherapy and 69 patients completed three cycles of chemotherapy. After induction chemotherapy, one patient received salvage surgery and eight were not given radiotherapy due to their poor general condition. Therefore, 65 patients (88%) received CRT. Based on genotype, HPV + was detected in 21 patients (28%), while 35 patients (47%) showed p16+ expression by IHC analysis. In the HPV genotype analysis, 16 patients (76%) were positive for genotype 16, 6 (29%) for genotype 18 and one each for genotypes 33 and 35. Among these, three patients were positive for both genotypes 16 and 18. The correlation between HPV + and p16+ expression was statistically significant (*p* <0.001). Tumor site and HPV status were not related. Among the characteristics of the patients, smoking history (never smoking) and performance status (PS; PS 0) were significantly associated with HPV and p16 (Table 1).

**Table 1:** **Patient and clinicopatholgic characteristics**

Patient demographics	Total *n*(%)	HPV status, *n*(%)			p16 status, *n*(%)		
		Positive	Negative	*p*	Positive	Negative	*p*
Age (Y, median)				<0.001			<0.001
≥70Y	70	56	65		57	68	
Range	43-80	43-77	43-80		43-77	48-80	
Gender				0.135			0.147
Male	69 (93)	18 (86)	51 (96)		31 (89)	38 (97)	
Female	5 (7)	3 (14)	2 (4)		4 (11)	1 (3)	
Alcohol				0.366			0.292
Never/ social	56 (76)	17 (81)	39 (74)		28 (80)	28 (72)	
Heavy	18 (24)	4 (19)	14 (26)		7 (20)	11 (28)	
Anatomic site				0.376			0.692
Tonsil	47 (64)	15 (71)	32 (60)		24 (69)	23 (59)	
Soft palate	10 (13)	1 (5)	9 (17)		4 (11)	6 (15)	
Tongue base	17 (23)	5 (24)	12 (23)		7 (20)	10 (26)	
Clinical stage				0.366			0.502
III	18 (24)	4 (24)	14 (26)		9 (26)	9 (23)	
IV	56 (76)	56 (76)	39 (74)		26 (74)	30 (77)	
T stage				0.181			0.062
T1-2	45 (61)	15 (71)	30 (57)		25 (71)	20 (51)	
T3-4	29 (39)	6 (29)	23 (43)		10 (29)	19 (49)	
N stage				0.305			0.601
N0-1	19 (26)	4 (19)	15 (28)		9 (26)	10 (26)	
N2-3	55 (74)	17 (81)	38 (72)		26 (74)	29 (74)	
Differentiation				0.346			0.099
Well	23 (31)	4 (19)	19 (36)		14 (40)	10 (26)	
Moderate	19 (26)	6 (29)	13 (25)		6 (17)	17 (44)	
Poorly	8 (11)	4 (19)	4 (8)		10 (29)	9 (23)	
NA	24 (32)	7 (33)	17 (32)		5 (14)	3 (7)	
PS				0.007			0.001
0	42 (57)	17 (81)	25 (47)		27 (77)	15 (38)	
1	32 (43)	4 (19)	28 (53)		8 (23)	24 (62)	
HPV							<0.001
Positive	21 (28)	21 (100)	0		21 (60)	0	
Negative	53 (72)	0	53 (100)		14 (40)	39 (100)	
p16				<0.001			
Positive	35 (47)	21 (100)	0		35 (100)	0	
Negative	39 (53)	0	0		0	39 (100)	
Induction response				0.436			0.018
CR	22 (30)	7 (33)	15 (28)		15 (43)	7 (18)	
Non-CR	52 (70)	14 (67)	38 (72)		20 (57)	32 (82)	
RT dose (cGy)				0.429			0.470
≥6,500	43 (58)	6 (67)	37 (57)		21 (60)	22 (56)	
<6,500	31 (42)	3 (34)	28 (43)		14 (40)	17 (44)	
RT type				0.070			0.289
3D-RT	52 (80)	5 (56)	47 (84)		25 (76)	27 (84)	
IMRT	13 (20)	4 (44)	9 (16)		8 (24)	5 (16)	
Treatment duration				0.501			0.092
<4 months	29 (39)	3 (33)	26 (40)		17 (49)	12 (31)	
≥4 months	45 (61)	6 (67)	39 (60)		18 (51)	27 (69)	

*Abbreviation*: *CR,* complete response.

### Expression of p53, class III beta-tubulin, ERCC1 and bcl-2 differed according to HPV status

p53, class III beta-tubulin, bcl-2 and ERCC1 expression were not correlated with clinical stage, with the exception of a trend towards an early T stage in p16+ patients (*p* = 0.062). However, p53 and class III beta-tubulin expression were significantly higher in HPV- and p16- than HPV + and p16+ patients (Table 2). Conversely, bcl-2 expression was greater in HPV + or p16+ than HPV- or p16- patients. ERCC1 expression did not differ significantly according to HPV status. There was no significant difference between HPV genotype and protein expression. Non-smokers showed more frequent HPV + (*p* = 0.047) and p16+ (*p* = 0.005) than smokers. However, expression of p53, ERCC1, and class III beta-tubulin were not associated with smoking history.

**Table 2:** **The expression of p53, ERCC1, beta-tubulin and bcl-2 stratified by HPV and p16 status**

	Total *n*(%)	HPV status, *n*(%)			p16 status, *n*(%)		
		Positive	Negative	*p*	Positive	Negative	*p*
p53				<0.001			<0.001
Low	49 (66)	20 (95)	29 (55)		32 (91)	17 (44)	
High	25 (34)	1 (5)	24 (45)		3 (9)	22 (56)	
ERCC1				0.329			0.422
Low	34 (46)	11 (52)	23 (43)		17 (49)	17 (44)	
High	40 (54)	10 (48)	30 (57)		18 (51)	22 (56)	
Class III beta tubulin				0.002			0.001
Low	52 (70)	20 (95)	32 (60)		31 (89)	21 (54)	
High	22 (30)	1 (5)	21 (40)		4 (11)	18 (46)	
Bcl-2				0.003			0.006
Low	57 (77)	11 (52)	46 (87)		22 (63)	35 (90)	
High	17 (23)	10 (48)	7 (13)		13 (37)	4 (10)	

### Treatment outcome and survival according to CRT response and protein status

**Figure 1:**
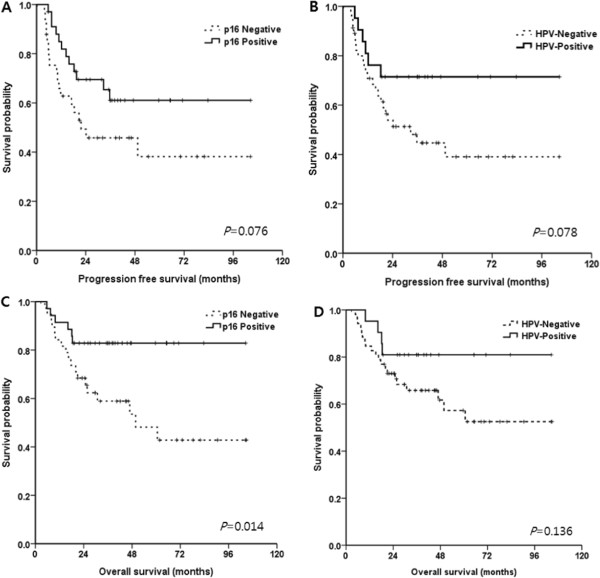
**PFS and OS according to HPV and p16 expression.** The 3Y-PFS showed a greater trend in p16+ or HPV+ than p16- or HPV- patients **(A, B)**. In the OS analysis, the 3Y-OS in p16+ patients was significantly higher than in p16- patients **(C)**. However, there was no statistical significance according to HPV status **(D)**.

A CR to induction chemotherapy was observed in 22 patients (30%) and a partial response in 46 patients (62%). Subsequent CRT after induction chemotherapy was performed in 65 patients. Among them, 37 patients (57%) showed CR and 6 patients (9%) who showed partial response received salvage operation. The CR rate after induction chemotherapy was significantly higher in p16+ (43%) than p16- (18%) patients (*p* = 0.018), and HPV was not associated significantly with the CR rate. There was no significant difference in chemotherapy response according to HPV genotypes. Patients who did not show a CR after induction chemotherapy tended to express a higher level of p53 than CR patients regardless of HPV status (*p* = 0.055); however, expression of ERCC1, class III beta-tubulin and bcl-2 were not associated significantly with a CR. During follow-up (median 48.3 months) period, 30 patients had progressive disease and 24 died. The median PFS and OS were not reached at the time of analysis. The 3Y-PFS and 3Y-OS rate were 53.4 ± 6.5% (95% CI, 40.66 - 66.14) and 70.3 ± 5.5% (95% CI, 59.52 - 81.08), respectively. 3Y-PFS showed a greater trend in HPV + (*p* = 0.078 Figure 1B) or p16+ (*p* = 0.076 Figure 1A) than HPV- or p16- patients. In the OS analysis, the 3Y-OS in p16+ patients was significantly higher (82.9%, 95% CI 70.36 - 95.44) than in p16- patients (58.9%, 95% CI 42.63 - 75.17, *p* = 0.014, Figure 1C, D).

**Figure 2:**
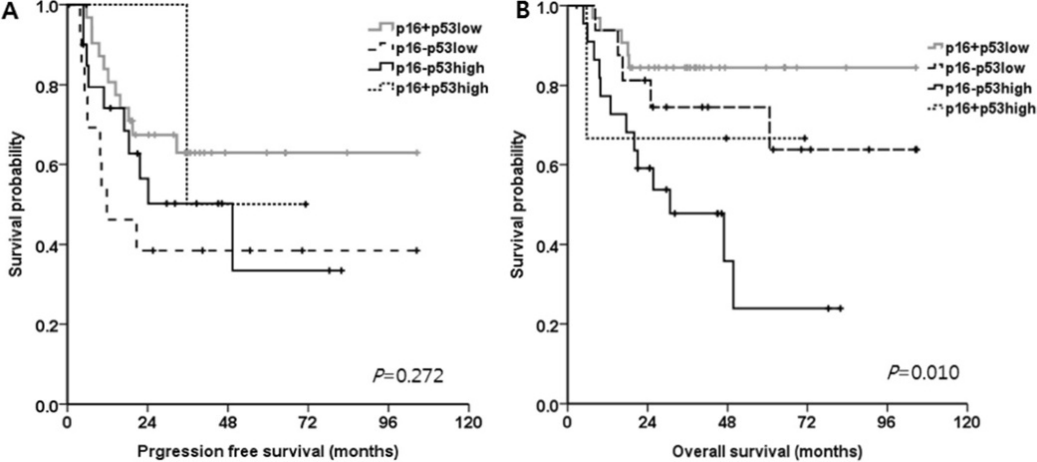
**PFS and OS based on p16 positivity and p53 expression.** PFS was not different according to p16 positivity and p53 expression **(A)**. OS was significantly greater in p16+/p53 low expression patients than other groups **(B)**.

In univariate analyses, clinical features such as an early T stage (T1-2 rather than T3-4) and good PS (PS 0) were significantly associated with the 3Y-PFS rate. In addition, PS (PS 0) and achievement of CR, completion of RT (≥6,500 cGy) showed a significantly better 3Y-OS rate than the other OS rates (Table 3). The expression of p53, ERCC1, class III beta-tubulin and bcl-2 did not influence the 3Y-PFS rate. However, p16, low p53 (*p* = 0.002) and class III beta-tubulin (*p* = 0.012) expression were significantly related to a higher 3Y-OS rate than was high expressions. In multivariate analyses, early T stage (*p* = 0.036) and PS 0 (*p* = 0.029) showed a better 3Y-PFS rate compared to late T stage or PS 1. In the 3Y-OS rate, low p53 expression (*p* = 0.012) and a CR (*p* = 0.026) were significant prognostic factors for the 3Y-OS rate (Table 4). When the patients were divided into four groups according to p16 positivity and p53 expression (low and high), the OS was significantly greater in p16+/p53 low expression patients than other groups (*p* = 0.010, Figure 2B).

**Table 3:** **Univariate analysis for survival**

	3Y-PFS, % (95% CI)	*p*	3Y-OS % (95% CI)	*p*
Gender		0.301		0.571
Male	51 (37.7-64.3)		70 (58.3-80.7)	
Female	80 (43.0-117.0)		80 (44.9-115.1)	
Smoking		0.793		0.642
Never	50 (28.2-71.4)		73 (55.4-89.9)	
Ex/current	55 (39.7-71.3)		69 (55.7-82.7)	
Alcohol		0.217		0.338
Never/social	56 (42.0-70.6)		72 (59.7-84.1)	
Heavy	45 (21.0-69.6)		66 (44.1-88.4)	
Anatomic site		0.235		0.563
Tonsil	59 (43.5-73.7)		71 (57.7-84.4)	
Others	44 (21.6-65.8)		69.2 (51.4-87.0)	
Clinical stage		0.952		0.930
III	54 (39.4-68.8)		72 (53.3-92.3)	
IV	53 (38.5-67.5)		61 (42.7-78.7)	
T stage		0.005		0.710
T1-2	63 (47.7-79.1)		77 (64.1-89.5)	
T3-4	37 (17.6-56.8)		61 (42.7-78.7)	
N stage		0.750		0.805
N0-1	54 (39.4-68.8)		73 (53.3-93.3)	
N2-3	51 (25.8-76.8)		69 (56.3-81.7)	
PS (ECOG)		0.004		0.003
0	62 (44.3-80.0)		85 (73.3-96.1)	
1	26 (5.5-47.5)		52 (34.4-69.6)	
Induction response		0.549		0.002
CR	53 (29.2-75.8)		94 (91.8-105.8)	
Non-CR	54 (39.2-69.0)		61 (47.2-74.2)	
RT dose (cGy)		0.233		0.006
≥6,500	57 (41.6-73.0)		80 (67.5-92.5)	
<6,500	46 (24.7-67.1)		56 (38.4-73.6)	
RT type		0.132		0.198
3D-RT	59 (42.5-67.8)		74 (62.0-86.4)	
IMRT	73 (50.5-92.5)		88.1 (78.1-98.1)	
Treatment duration		0.702		0.258
<4 months	59 (36.3-81.0)		75 (58.4-91.0)	
≥4 months	53 (36.6-68.8)		67 (53.3-81.5)	
HPV		0.078		0.136
Positive	71 (52.0-90.8)		81 (64.1-98.0)	
Negative	45 (29.0-60.4)		66 (52.5-79.1)	
p16		0.076		0.014
Positive	61 (43.4-78.6)		83 (70.4-95.4)	
Negative	46 (28.2-63.4)		59 (42.6-75.2)	
p53		0.655		0.002
Low	43 (25.5-71.7)		81 (69.8-92.2)	
High	49 (28.0-58.2)		50 (30.1-70.5)	
ERCC1		0.491		0.355
Low	53 (31.2-74.4)		64 (48.3-78.9)	
High	54 (26.8-82.0)		70 (41.3-99.3)	
Class III beta tubulin		0.098		0.012
Low	58 (43.1-72.5)		77 (65.4-89.4)	
High	42 (18.4-66.2)		54 (32.5-74.9)	
Bcl-2		0.678		0.217
Low	57 (40.6-72.8)		64 (48.3-78.9)	
High	54 (26.8-82.0)		70 (41.2-99.3)	

*Abbreviation*: *CR* complete response.

**Table 4:** **Multivariate analysis for survival**

	3Y PFS HR (95% CI)	*p*	3Y-OS HR (95% CI)	*p*
T (1–2 vs. 3–4)	2.218 (1.054-4.668)	0.036	-	
PS (ECOG 0 vs. 1)	2.341 (1.093-5.017)	0.029		
p53 (low vs. high)	-		2.863 (1.261-6.500)	0.012
Postinduction CR (CR vs. non-CR)	-		9.847 (1.322-73.372)	0.026

*Abbreviation*: *CR* complete response.

## Discussion

This is the first report of differential expression of biomarkers between HPV OPC and non-HPV OPC under a single treatment strategy. HNSCC associated with OPC has a higher survival rate than non- OPC-associated HNSCC [4]; however, comparisons of biomarkers based on HPV status are lacking. In the current study we determined the protein expression profiles associated with OPC treatment outcomes. Radiotherapy is an important head-and-neck cancer treatment modality, but measuring the response is difficult due to radiation-induced tissue swelling. Therefore, we selected patients treated with induction chemotherapy. To avoid bias due to use of different chemotherapy regimens, the induction regimen was limited to a combination chemotherapy using DCF.

Previous studies reported the prognostic role of biomarkers in head and neck cancers [17, 18, 25]. Among them, p53 is a well-known biomarker for various tumors, including head-and-neck cancers [17]. In HPV OPC, p53 is usually not mutated, but its expression is low due to HPV E6 protein activity, which targets p53 for ubiquitination and degradation [9]. This phenomenon may preserve the apoptotic function of p53 even though its level is low, and thus enable radiation-induced apoptosis [26]. The p53 status is an important factor in the treatment response and may indicate different treatment outcomes among HNSCC patients, including those with OPC [12, 16]. Reportedly, p53 missense mutation increases protein stability, which is easily detected by IHC [17]. In addition, beta tubulin has been investigated extensively in patients treated with taxane for various types of cancer [20, 25, 27]. In the TAX 324 study, class II beta-tubulin expression was examined in locally advanced head-and-neck cancer (LAHNC) patients treated with induction chemotherapy with DCF or 5-FU with cisplatin [25]. It suggested that high expression of class II beta tubulin showed adverse treatment outcome after induction chemotherapy. On the other hand, we reported the predictive role of class III beta-tubulin in gastric cancer treated with taxane-based chemotherapy [20]; a similar finding was reported in LAHNC patients [18]. Based on this premise, we evaluated class III beta-tubulin in this study.

In the present study, HPV OPC occurred frequently in young adults, non-smokers and good PS patients. However, the clinical characteristics and protein expression associated with chemotherapy response differed significantly according to HPV status. p53 and class III beta-tubulin were more highly expressed in non-HPV OPC than HPV OPC patients. However, the relationship between p53 and smoking was not established in this study, and could be related to the medical records depend on patient history. Low p53 expression was significantly correlated with a CR to induction chemotherapy, and was a good prognostic factor in the multivariate analysis. Therefore, p53 had high prognostic value for OPC. Interestingly, three patients showed p53 high/p16+, and they showed inferior 3Y-PFS and 3Y-OS than p53 low/p16+ patients. It means that the pathogenesis could be combined with HPV and other factors. Therefore, although the number is small, p16 positivity itself could be lacking to predict the treatment outcome in OPC. Class III beta-tubulin was also highly expressed in non-HPV patients and was associated with a poor 3Y-OS. The anti-apoptotic factor bcl-2 was more highly expressed in HPV-OPC than non-HPV OPC patients, and had no effect on chemotherapy response or survival. In general, high bcl-2 expression shows a poor response to chemotherapy or radiotherapy in solid tumors [28–30]. However, reports regarding high bcl-2 expression predicting a good response in HNSCC [21, 25] or lung cancer [31] are conflicting. To define the role of bcl-2, we analyzed bcl-2 expression according to HPV status. The OS in p16+ patients did not differ according to the bcl-2 expression level (83.4 months for low expression, 78.4 months for high expression). In contrast, although the number of patients was insufficient to achieve statistical significance, low bcl-2 expression (63.2 months) showed a trend to a better OS compared to high bcl-2 expression (20.5 months) in p16- patients. This result suggested that although bcl-2 was highly expressed in p16+ OPC patients, other mechanisms (e.g., lower p53 or class III beta-tubulin expression) might act individually or in concert to modulate proapoptosis when exposed to chemotherapy and overcome chemoresistance of bcl-2. However, in p16- patients, bcl-2 has an anti-apoptotic function with strong expression of p53 and class III beta tubulin. Confirming the role of bcl-2 in various types of cancer of heterogeneous causes is difficult; therefore, further studies with larger cohorts that include other biomarkers are necessary to define the role of bcl-2.

In multivariate analysis, PFS was associated with clinical characteristics such as early T stage and good PS score, rather than biochemical markers. However, p53 and class III beta-tubulin expression levels were significant prognostic factors in terms of OS. CR after induction chemotherapy was also associated with good survival, and patients who showed a CR tended to express lower levels of p53 compared to non-CR patients, suggesting the important factors associated with prognosis are related to biological characteristics other than tumor stage. This may be a reasonable basis for de-escalation treatment trials in HPV OPC patients. Regardless of HPV status, completion of radiotherapy is an important prognostic factor for OS in univariate analysis (*p* = 0.006).

To investigate the effects of HPV in OPC, an optimal detection method is important. In a clinical setting, specificity, sensitivity and convenience are necessary for treatment planning. In terms of sources, paraffin-embedded tissue is more easily obtained than fresh tissue. The direct detection methods for HPV infection are polymerase chain reaction (PCR) and *in situ* hybridization (ISH). Both are highly specific for detection of HPV infection, but PCR does not distinguish the presence of transcriptionally inactive viral DNA or enable identification of cross-contamination of samples that may lead to false-positive results [32]. ISH assays capable of detecting multiple high-risk HPV types have lower sensitivity than PCR. IHC staining for p16 has been substituted for HPV PCR or ISH for HPV detection because p16 overexpression indicated the presence of active and functional viral oncoprotein and is readily detected in paraffin-embedded tissue. In the present study, all HPV + patients (n = 28) were p16+; however, 19 patients were p16+ but HPV- based on PCR. In survival subgroup analysis, the survival outcomes of HPV+/p16+ and HPV-/p16+ patients showed similar trends contrast to HPV-/p16- patients. The discrepancy between p16+ and HPV PCR- patients may be associated with functional pRb disturbances unrelated to the HPV infection (e.g., mutational inactivation of Rb protein) [33, 34]. Therefore, p16 is a potential surrogate marker for the prognosis of OPC presenting of HPV infection in addition to show its functional status and easy availability.

The strength of the current study was the direct comparison of protein expression in OPC according to HPV status under the same treatment regimen, followed by CCRT. To date, several studies of OPC biomarkers using various chemotherapy regimens, radiotherapy or surgery [17, 18, 25], have been conducted, which makes clarification of the roles of the biomarkers problematic. In addition, we documented the crucial role of p53, which was differentially expressed according to HPV status, influenced on CR and survival. However, the present study has several limitations. We did not adjust for multiple comparisons, which might lead to Type 1 error in statistical analysis. And other biomarkers such as EGFR, Ku80, thymidylate synthase or glutathione s-transferase (GST) were not evaluated. Therefore, larger studies are needed to confirm our findings including these biomarkers.

## Conclusions

OPC showed distinct protein expression related with chemotherapy response according to HPV status. Low p53, class III beta-tubulin expression and high bcl-2 were expressed in HPV OPC compared to non-HPV OPC and these results suggest that better chemotherapy response of HPV OPC could be associated with different expression of these proteins. Among these biomarkers, p53 in addition to p16 could be used to design the individual treatment strategies for OPC.

## References

[1] Frisch M, Hjalgrim H, Jaeger AB, Biggar RJ (2000). Changing patterns of tonsillar squamous cell carcinoma in the United States. Cancer Causes Control.

[2] Nasman A, Attner P, Hammarstedt L, Du J, Eriksson M, Giraud G, Ahrlund-Richter S, Marklund L, Romanitan M, Lindquist D, Ramqvist T, Lindholm J, Sparén P, Ye W, Dahlstrand H, Munck-Wikland E, Dalianis T (2009). Incidence of human papillomavirus (HPV) positive tonsillar carcinoma in Stockholm, Sweden: an epidemic of viral-induced carcinoma?. Int J Cancer.

[3] Shin A, Jung YS, Jung KW, Kim K, Ryu J, Won YJ (2013). Trends of human papillomavirus-related head and neck cancers in Korea: national cancer registry data. Laryngoscope.

[4] Ang KK, Harris J, Wheeler R, Weber R, Rosenthal DI, Nguyen-Tan PF, Westra WH, Chung CH, Jordan RC, Lu C, Kim H, Axelrod R, Silverman CC, Redmond KP, Gillison ML (2010). Human papillomavirus and survival of patients with oropharyngeal cancer. N Engl J Med.

[5] Fakhry C, Westra WH, Li S, Cmelak A, Ridge JA, Pinto H, Forastiere A, Gillison ML (2008). Improved survival of patients with human papillomavirus-positive head and neck squamous cell carcinoma in a prospective clinical trial. J Natl Cancer Inst.

[6] Ragin CC, Taioli E (2007). Survival of squamous cell carcinoma of the head and neck in relation to human papillomavirus infection: review and meta-analysis. Int J Cancer.

[7] Robinson M, Sloan P, Shaw R (2010). Refining the diagnosis of oropharyngeal squamous cell carcinoma using human papillomavirus testing. Oral Oncol.

[8] Westra TA, Parouty M, Brouwer WB, Beutels PH, Rogoza RM, Rozenbaum MH, Daemen T, Wilschut JC, Boersma C, Postma MJ (2012). On discounting of health gains from human papillomavirus vaccination: effects of different approaches. Value Health.

[9] Chung CH, Gillison ML (2009). Human papillomavirus in head and neck cancer: its role in pathogenesis and clinical implications. Clin Cancer Res.

[10] Braakhuis BJ, Snijders PJ, Keune WJ, Meijer CJ, Ruijter-Schippers HJ, Leemans CR, Brakenhoff RH (2004). Genetic patterns in head and neck cancers that contain or lack transcriptionally active human papillomavirus. J Natl Cancer Inst.

[11] Butz K, Geisen C, Ullmann A, Spitkovsky D, Hoppe-Seyler F (1996). Cellular responses of HPV-positive cancer cells to genotoxic anti-cancer agents: repression of E6/E7-oncogene expression and induction of apoptosis. Int J Cancer.

[12] Bristow RG, Benchimol S, Hill RP (1996). The p53 gene as a modifier of intrinsic radiosensitivity: implications for radiotherapy. Radiother Oncol.

[13] Olthof NC, Straetmans JM, Snoeck R, Ramaekers FC, Kremer B, Speel EJ (2012). Next-generation treatment strategies for human papillomavirus-related head and neck squamous cell carcinoma: where do we go?. Rev Med Virol.

[14] Vermorken JB, Remenar E, Van Herpen C, Gorlia T, Mesia R, Degardin M, Stewart JS, Jelic S, Betka J, Preiss JH, van den Weyngaert D, Awada A, Cupissol D, Kienzer HR, Rey A, Desaunois I, Bernier J, Lefebvre JL, EORTC 24971/TAX 323 Study Group (2007). Cisplatin, fluorouracil, and docetaxel in unresectable head and neck cancer. N Engl J Med.

[15] Posner MR, Hershock DM, Blajman CR, Mickiewicz E, Winquist E, Gorbounova V, Tjulandin S, Shin DM, Cullen K, Ervin TJ, Murphy BA, Raez LE, Cohen RB, Spaulding M, Tishler RB, Roth B, Viroglio Rdel C, Venkatesan V, Romanov I, Agarwala S, Harter KW, Dugan M, Cmelak A, Markoe AM, Read PW, Steinbrenner L, Colevas AD, Norris CM, Haddad RI, TAX 324 Study Group (2007). Cisplatin and fluorouracil alone or with docetaxel in head and neck cancer. N Engl J Med.

[16] Temam S, Flahault A, Perie S, Monceaux G, Coulet F, Callard P, Bernaudin JF, St Guily JL, Fouret P (2000). p53 gene status as a predictor of tumor response to induction chemotherapy of patients with locoregionally advanced squamous cell carcinomas of the head and neck. J Clin Oncol.

[17] Shinohara S, Kikuchi M, Tona R, Kanazawa Y, Kishimoto I, Harada H, Imai Y, Usami Y (2014). Prognostic impact of p16 and p53 expression in oropharyngeal squamous cell carcinomas. Jpn J Clin Oncol.

[18] Koh Y, Kim TM, Jeon YK, Kwon TK, Hah JH, Lee SH, Kim DW, Wu HG, Rhee CS, Sung MW, Kim CW, Kim KH, Heo DS (2009). Class III beta-tubulin, but not ERCC1, is a strong predictive and prognostic marker in locally advanced head and neck squamous cell carcinoma. Ann Oncol.

[19] Olaussen KA, Dunant A, Fouret P, Brambilla E, Andre F, Haddad V, Taranchon E, Filipits M, Pirker R, Popper HH, Stahel R, Sabatier L, Pignon JP, Tursz T, Le Chevalier T, Soria JC, IALT Bio Investigators (2006). DNA repair by ERCC1 in non-small-cell lung cancer and cisplatin-based adjuvant chemotherapy. N Engl J Med.

[20] Hwang JE, Hong JY, Kim K, Kim SH, Choi WY, Kim MJ, Jung SH, Shim HJ, Bae WK, Hwang EC, Lee KH, Lee JH, Cho SH, Chung IJ (2013). Class III beta-tubulin is a predictive marker for taxane-based chemotherapy in recurrent and metastatic gastric cancer. BMC Cancer.

[21] Pena JC, Thompson CB, Recant W, Vokes EE, Rudin CM (1999). Bcl-xL and Bcl-2 expression in squamous cell carcinoma of the head and neck. Cancer.

[22] Greene FL (2002). The American joint committee on cancer: updating the strategies in cancer staging. Bull Am Coll Surg.

[23] Romero-Pastrana F (2012). Detection and typing of human papilloma virus by multiplex PCR with type-specific primers. ISRN Microbiol.

[24] Begum S, Gillison ML, Ansari-Lari MA, Shah K, Westra WH (2003). Detection of human papillomavirus in cervical lymph nodes: a highly effective strategy for localizing site of tumor origin. Clin Cancer Res.

[25] Cullen KJ, Schumaker L, Nikitakis N, Goloubeva O, Tan M, Sarlis NJ, Haddad RI, Posner MR (2009). Beta-Tubulin-II expression strongly predicts outcome in patients receiving induction chemotherapy for locally advanced squamous carcinoma of the head and neck: a companion analysis of the TAX 324 trial. J Clin Oncol.

[26] Peltenburg LT (2000). Radiosensitivity of tumor cells. Oncogenes and apoptosis. Q J Nucl Med.

[27] Zhang HL, Ruan L, Zheng LM, Whyte D, Tzeng CM, Zhou XW (2012). Association between class III beta-tubulin expression and response to paclitaxel/vinorebine-based chemotherapy for non-small cell lung cancer: a meta-analysis. Lung Cancer.

[28] Real PJ, Sierra A, De Juan A, Segovia JC, Lopez-Vega JM, Fernandez-Luna JL (2002). Resistance to chemotherapy via Stat3-dependent overexpression of Bcl-2 in metastatic breast cancer cells. Oncogene.

[29] Kamesaki S, Kamesaki H, Jorgensen TJ, Tanizawa A, Pommier Y, Cossman J (1993). Bcl-2 protein inhibits etoposide-induced apoptosis through its effects on events subsequent to topoisomerase II-induced DNA strand breaks and their repair. Cancer Res.

[30] Nix P, Cawkwell L, Patmore H, Greenman J, Stafford N (2005). Bcl-2 expression predicts radiotherapy failure in laryngeal cancer. Br J Cancer.

[31] Jeong SH, Jung JH, Han JH, Kim JH, Choi YW, Lee HW, Kang SY, Hwang YH, Ahn MS, Choi JH, Oh YT, Chun M, Kang S, Park KJ, Hwang SC, Sheen SS (2010). Expression of Bcl-2 predicts outcome in locally advanced non-small cell lung cancer patients treated with cisplatin-based concurrent chemoradiotherapy. Lung Cancer.

[32] Ritchie JM, Smith EM, Summersgill KF, Hoffman HT, Wang D, Klussmann JP, Turek LP, Haugen TH (2003). Human papillomavirus infection as a prognostic factor in carcinomas of the oral cavity and oropharynx. Int J Cancer.

[33] Gronhoj Larsen C, Gyldenlove M, Jensen DH, Therkildsen MH, Kiss K, Norrild B, Konge L, Von Buchwald C (2014). Correlation between human papillomavirus and p16 overexpression in oropharyngeal tumours: a systematic review. Br J Cancer.

[34] Marur S, D’Souza G, Westra WH, Forastiere AA (2010). HPV-associated head and neck cancer: a virus-related cancer epidemic. Lancet Oncol.

[CR35] The pre-publication history for this paper can be accessed here: http://www.biomedcentral.com/1471-2407/14/824/prepub

